# Supplementary material for: Oocyte-Specific Knockout of Histone Lysine Demethylase KDM2a Compromises Fertility by Blocking the Development of Follicles and Oocytes
Source: Int J Mol Sci. 2022 Oct 9;23(19):12008. doi: 10.3390/ijms231912008 (PMC9570323; doi:10.3390/ijms231912008)
Supplement: Supplementary file 1 [file ijms-23-12008-s001.zip › ijms-1934578-supplementary.pdf]

Supplementary

Table S1 primers used for genotyping and RT-PCR of *Kdm2a*$^{flox/flox}$ mice

Gene name	Sequence (5'-3')	Product size/bp
Floxed *Kdm2a*	TCACCCTTGCAACTTCCTTCTTCCTT<br>TGCACACTGACAAACTCAGCTACCA	Wild type: 397<br>Floxed: 510
Zp3-Cre	CATATTGGCAGAACGAAAACGC<br>CATATTGGCAGAACGAAAACGC	413
Kdm2a	GCCAAGGCACTTGAAAGAAA<br>AGCAGCCTCGAACACTCATT	104
Actb	AAGATCATTGCTCCTCCTGAGC<br>CCTACTCCTGCTTGCTGATCCA	106

RT-PCR, real-time PCR; Zp3, zona pellucida protein 3, *Actb*, beta-actin.

Table S2 The sequences of primers and gene accession number used in RT-qPCR.

Gene	Primer sequence	Gene accession number
Fshr	Fw: GAGGGCCAGGTCAACATACC<br>Rs: GAAGTTCAGAGGTTTGCCGC	NM_013523.3
Amh	Fw: CTGGCTAGGGGAGACTGGAG<br>Rs: TCGGGCTCCCATATCACTTC	NM_007445.3
Gdf9	Fw: CAACCAGGTGACAGGACCG<br>Rs: CACCCGGTCCAGGTTAAACA	NM_008110.2
Inha	Fw: TATTCCGGCCATCCCAACAC<br>Rs: CAGAAGATCTAGCAGGGGCG	NM_010564.5
Bmp15	Fw: AAGGGAGAACCGCACGATTG<br>Rs: TGTACATGCCAGGAACCTCTG	NM_009757.5
Star	Fw: TAAACTCACTTGGCTGCT<br>Rs: GGTGGTTGGCGAACTC	NM_011485.5
Cyp19a1	Fw: TCTCGATTCGGCAGCAAACT<br>Rs: CGTCCACATAGCCCGATTCA	NM_007810.3
Kdm2b	Fw: CCCTGATTTCACAGTCCG<br>Rs: GTTGTACAGTTTATCCCGCT	NM_001003953.2

Lhcgr	Fw: CTCGCCCGACTATCTCTCAC<br>Rs: TTGAGGAGGTTGTCAAAGGCA	NM_013582.3
Actb	Fw: AAGATCATTGCTCCTCCTGAGC<br>Rs: CCTACTCCTGCTTGCTGATCCA	NM_007393.3

Fw: forward, Rs: reverse.

Table S3 Evaluation of the reproductive function of Kdm2a cKO females

Mating program	Mating pairs	Total Pups	Pups/ Pair	Total Litters	Litters/ Pair	Pups/ Litters
♀cKO x ♂WT	6	91b	15.2 ± 1.5b	19	3.2 ± 1.0	4.8 ± 1.2b
♀Con x ♂WT	6	175a	29.2 ± 1.1a	18	3.0 ± 0.8	9.7 ± 0.9a

The breeding pairs with the indicated genotypes were housed together for 4 months and the reproductive data of each breeding pair were recorded. Values with dissimilar superscripts (a-c) in the same column are significantly different ($P < 0.05$). Con: *Kdm2a*$^{flox/flox}$.

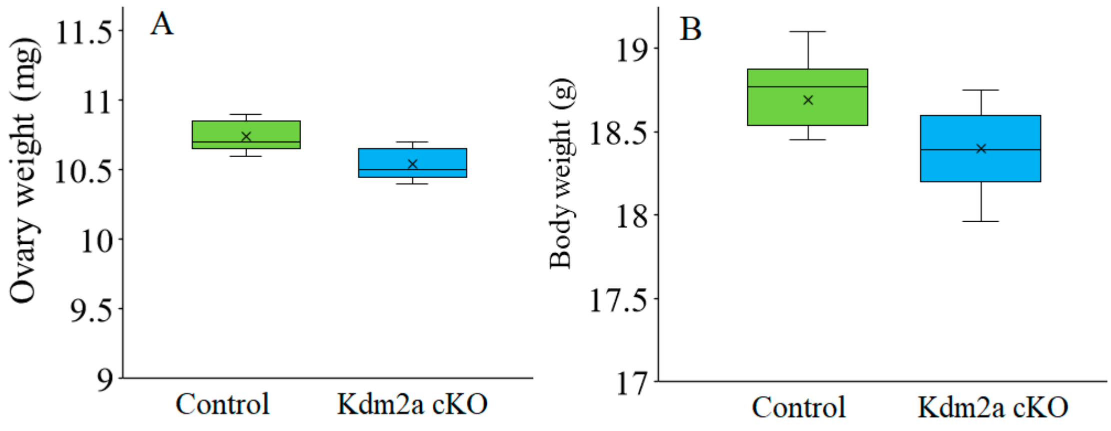

Figure S1. *Kdm2a* cKO ovaries and body display no significant difference. (A) The ovary weight of 4-week-old from control and *Kdm2a* cKO mice (n=8). (B) The body weight of 4-week-old from control and *Kdm2a* cKO mice (n=10).

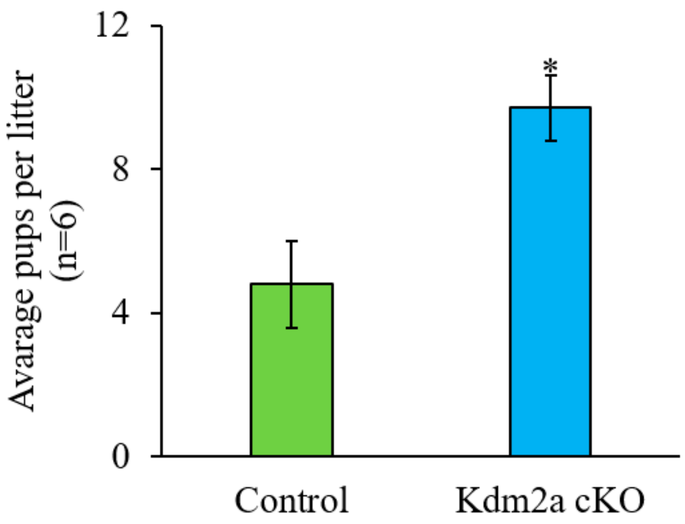

Figure S2. Effect of *Kdm2a* knockout on the reproduction. The avarage number of pups produced from control and *Kdm2a* cKO group. * Significant difference ($P <$ 0.05).
